# RNA methylation preserves ES cell identity by chromatin silencing of retrotransposons

**DOI:** 10.1038/s41392-021-00683-4

**Published:** 2021-07-07

**Authors:** Zhaofa Xu, Annabel Ma, Yongchao C. Ma

**Affiliations:** Departments of Pediatrics, Neurology and Physiology, Northwestern University Feinberg School of Medicine, Ann & Robert H. Lurie Children’s Hospital of Chicago, Chicago, IL USA

**Keywords:** Epigenetics, Embryonic stem cells

RNA methylation on N^6^-methyladenosine (m^6^A) and histone methylation are critical mechanisms regulating gene expression at epitranscriptomic and epigenetic levels, respectively. However, how RNA methylation modulates histone methylation, and in which biological processes, is unknown. In a recent study published in *Nature*, Liu et al.^1^ identified the critical roles of m^6^A and its reader YTHDC1 in mediating histone methylation and chromatin silencing of retrotransposon genes to preserve embryonic stem (ES) cell identity.

During mouse embryonic development, the quiescent zygotic genome is activated during the first cell division, coinciding with the gain of totipotency.^2^ The transient totipotency at the two-cell stage will be quickly lost as embryos progress into blastocysts and later stages of development. Mouse ES cells derived from the blastocyst are pluripotent, so they can only give rise to embryonic, but not extra-embryonic (placental), cell types. ES cells in culture can transiently adopt a two-cell-like (2C-like) totipotent state. These 2C-like cells represent a good model for studying totipotency and ES cell identity regulation.

Methylation of RNA on N6-adenosine (m^6^A) is emerging as a critical mechanism regulating RNA metabolism and function.^3^ In mammals, methyltransferases METTL3 (methyltransferase-like 3) and METTL14 mediate the addition of m^6^A to target RNAs. Readers, such as YTH (YT521-B homology) domain proteins, bind and interpret m^6^A modification. Genetic knockout (KO) of methyltransferase *Mettl3* in mice leads to early embryonic lethality. Interestingly, genetic deletion of m^6^A reader *YTHDC1*, but not other YTH readers, shows similar phenotypes as *Mettl3*KO, suggesting that YTHDC1 may mediate the effects of losing m^6^A in *Mettl3*KO. To test this possibility, the authors generated *Ythdc1* conditional KO mouse ES cells with inducible CreERT and demonstrated that *Ythdc1* was required for ES cell maintenance. Surprisingly, RNA sequencing of *Ythdc1*KO ES cells showed upregulations of transposable elements (TEs) and 2C-like state markers. In addition, expression of wild-type *Ythdc1*, but not YTH m^6^A-binding-site mutants, repressed the activation of the 2C-like program, indicating the dependence on m^6^A. Together, these data suggest that YTHDC1 relies on its m^6^A binding to maintain ES cell identity and inhibit the transition to the 2C-like state.

To identify YTHDC1-binding methylated RNAs, the authors performed YTHDC1 RNA immunoprecipitation sequencing (RIP-seq) and m^6^A RIP-seq. Intriguingly, YTHDC1-binding peaks were preferentially located at TE loci, which were consistently m^6^A marked. TEs are mobile genetic elements whose sequences constitute nearly half of the human genome. Retrotransposons, representing the vast majority of TEs, include endogenous retroviral elements and long interspersed nuclear element 1 (LINE1). These two families of TEs are activated specifically at the 2C-like state and must be repressed for the transition into later stages of embryonic development. Indeed, the authors found that among the YTHDC1 target RNA differentially expressed upon *Ythdc1*KO, all 62 retrotransposon RNAs were upregulated. Interestingly, the genomic locations of these upregulated transcripts showed significant enrichment of chromatin silencing H3K9me3, suggesting that YTHDC1 represses retrotransposons via H3K9me3. One H3K9me3 methyltransferase that was shown by the authors’ group to target on retrotransposon regions to inhibit their expression and 2C-like transition is SETDB1.

**Fig. 1:**
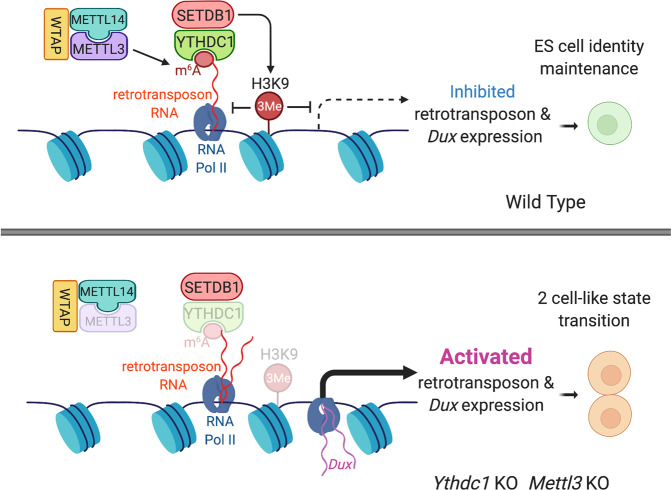
Working model for the mechanism underlying m6A and YTHDC1-mediated maintenance of embryonic stem cell identity through chromatin silencing. Methylation of retrotransposon RNA recruits m^6^A reader YTHDC1 and H3K9me3 methyltransferase SETDB1 to promote retrotransposon chromatin silencing, which represses two-cell-like state transition, leading to embryonic stem cell identity maintenance

Coimmunoprecipitation revealed physical interactions between YTHDC1 and SETDB1, suggesting that m^6^A-binding YTHDC1 recruits SETDB1 to methylate H3K9 at specific retrotransposons (Fig. 1). YTHDC1 and H3K9me3 chromatin immunoprecipitation sequencing showed that 12,933 out of 18,525 YTHDC1-binding peaks were also marked by H3K9me3. These YTHDC1-H3K9me3 co-bound peaks mainly localize to retrotransposons, whose expression increases upon depletion of either *Ythdc1* or *Setdb1*. Importantly, 4sUDRB-seq was used to show that *Ythdc1*KO enhanced nascent RNA transcription of target retrotransposons, but not through inhibiting RNA decay. Then chromatin isolation by RNA purification (ChIRP) sequencing was used to show that YTHDC1 was recruited onto retrotransposon chromatin regions via their corresponding RNAs, which was confirmed by global RNA interactions with DNA by deep sequencing.

To further examine the m^6^A-dependence, the authors deleted *Mettl3* methyltransferase in mouse ES cells. *Mettl3*KO abolished m^6^A modification on 25 of the 45 m^6^A-marked retrotransposon families. *Mettl3*KO also impaired SETDB1-mediated H3K9me3 modification of retrotransposons and increased their expression, largely overlapping with those upregulated upon *Ythdc1*KO, indicating that RNA methylation is required for YTHDC1-mediated retrotransposon chromatin silencing. Furthermore, they found transcription factor Dux, which is necessary and sufficient to drive ES cells to the 2C-like state, was upregulated in *Ythdc1*KO, *Setdb1*KO, and *Mettl3*KO ES cells. Knocking out *Dux* was sufficient to block the induction of the 2C-like program in *Ythdc1*KO ES cells, demonstrating the essential role of *Dux* in the 2C-like transition upon *Ythdc1*KO. In addition, *Dux*KO cells retained the ability to reactivate many retrotransposons together with dampened H3K9me3 upon *Ythdc1* depletion, indicating that Dux functions downstream to retrotransposons and SETDB1-mediated H3K9me3 modification.

The authors used rigorous genetic KOs in mouse ES cells together with novel sequencing approaches such as ChIRP, to elegantly show that m^6^A preserves ES cell identity and inhibits 2C-like state transition through YTHDC1- and SETDB1-mediated retrotransposon chromatin silencing. Histone methylation was recently shown to regulate m^6^A demethylase ALKBH5 expression,^4^ therefore the crosstalk between RNA methylation and histone modifications is emerging as an exciting field for more investigation. Interestingly, several related articles were published independently around the same time as this study. The other papers identified different YTHDC1-interacting proteins for mediating retrotransposon chromatin silencing,^5^ suggesting multiple mechanisms may have been evolved to ensure the tight and precise regulation of ES cell identity. However, many questions remain to be addressed. For example, are these mechanisms applicable to mRNA and other noncoding RNA? How are these findings different from the m^6^A-dependent METTL3- and YTHDC1-mediated decay of chromosome-associated *LINE1* RNA in regulating chromatin state and transcription? How is METTL3 specifically recruited to retrotransposon-derived RNA for methylation? Answering these questions will elucidate whether this new mode of chromatin regulation can be applied to broader biological processes, possibly provide insights into disease pathogenesis, and facilitate therapeutic development.
